# Loss of BOSS Causes Shortened Lifespan with Mitochondrial Dysfunction in *Drosophila*

**DOI:** 10.1371/journal.pone.0169073

**Published:** 2017-01-03

**Authors:** Ayako Kohyama-Koganeya, Mizuki Kurosawa, Yoshio Hirabayashi

**Affiliations:** Molecular Membrane Neuroscience, Brain Science Institute, RIKEN, Wako-shi, Saitama, Japan; CINVESTAV-IPN, MEXICO

## Abstract

Aging is a universal process that causes deterioration in biological functions of an organism over its lifetime. There are many risk factors that are thought to contribute to aging rate, with disruption of metabolic homeostasis being one of the main factors that accelerates aging. Previously, we identified a new function for the putative G-protein-coupled receptor, Bride of sevenless (BOSS), in energy metabolism. Since maintaining metabolic homeostasis is a critical factor in aging, we investigated whether BOSS plays a role in the aging process. Here, we show that BOSS affects lifespan regulation. *boss* null mutants exhibit shortened lifespans, and their locomotor performance and gut lipase activity—two age-sensitive markers—are diminished and similar to those of aged control flies. Reactive oxygen species (ROS) production is also elevated in *boss* null mutants, and their ROS defense system is impaired. The accumulation of protein adducts (advanced lipoxidation end products [ALEs] and advanced glycation end products [AGEs]) caused by oxidative stress are elevated in *boss* mutant flies. Furthermore, *boss* mutant flies are sensitive to oxidative stress challenges, leading to shortened lives under oxidative stress conditions. Expression of superoxide dismutase 2 (SOD2), which is located in mitochondria and normally regulates ROS removal, was decreased in *boss* mutant flies. Systemic overexpression of SOD2 rescued *boss* mutant phenotypes. Finally, we observed that mitochondrial mass was greater in *boss* mutant flies. These results suggest that BOSS affects lifespan by modulating the expression of a set of genes related to oxidative stress resistance and mitochondrial homeostasis.

## Introduction

Aging is a complex phenomenon with a multifactorial etiology. Several theories have been proposed to explain how endogenous and exogenous factors affect aging. Reduced dietary caloric intake is known to extend lifespan in a wide range of organisms including mammals and worms [1, 2]. Caloric restriction induces changes in metabolic response. Loss of fat mass is the most prominent metabolic change in response to restriction, and lipid homeostasis seems to be an important factor in regulating lifespan [3]. Concomitantly, caloric restriction reduces ROS generation [4]. Thus, the regulation of oxidant production and the ability of organisms to respond to oxidative stress are intricately linked to aging and lifespan [5, 6].

According to the free-radical theory of aging, ROS are predominantly byproducts of mitochondrial metabolism and are thought to be a risk factor for aging [7]. Thus, the regulation of mitochondrial mass and activity is very important. How, though, are mitochondrial mass and activity regulated? Although mitochondria possess their own genome (mtDNA), the majority of mitochondrial proteins are encoded by the nuclear genome, translated in the cytoplasm, and imported into mitochondria [8]. Therefore, mitochondrial mass and activity must be coordinated with nutrient availability. Indeed, starvation or exercise increases mitochondrial mass [9].

Here, we describe the functions of BOSS in the aging process. BOSS was originally identified as a ligand of the receptor SEVENLESS and required for the development of the R7 photoreceptor neuron in the compound eye of Drosophila melanogaster [10]. Then we previously discovered that BOSS possesses a critical metabolic function: maintaining energy homeostasis [11, 12]. Insulin signaling is disrupted in boss mutant flies. Furthermore, boss mutant flies are lean but hyperphagia. Because changes in metabolism are tightly linked with aging, we speculated that BOSS might play a role in aging. Thus, we examined the lifespan of *boss* mutant flies and various aging phenotypes of *boss* mutant flies. We discovered that *boss* mutants have a short lifespan. Interestingly, we found that aging processes were accelerated in *boss* mutant flies. In young *boss* mutant flies, locomotor activity and gut lipase activity were reduced. Furthermore, oxidative stress (ALEs and AGEs) and oxidative damage were enhanced in *boss* mutants. However, overexpression of SOD2 rescued *boss* mutant flies from oxidative damage and from exhibiting an aged phenotype. Our findings suggest that BOSS may play a central role in lifespan control through its involvement in mitochondrial function.

## Results

### *boss* mutant flies have shortened lifespan and mobility impairment

**Fig 1:**
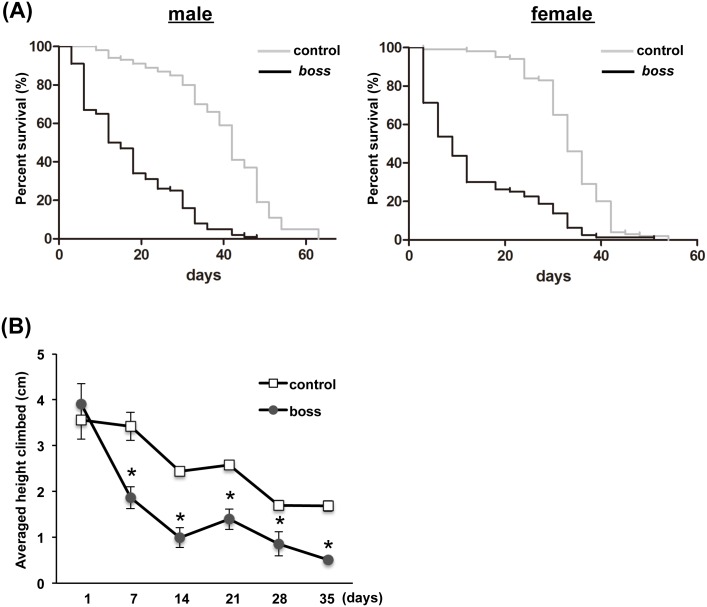
Aging is accelerated in *boss* mutant flies. (A) Lifespan for male and female *boss* mutant flies. *boss* mutant flies have shorter lifespan than control flies. Survival was presented by Kaplan Meier curves of control and *boss* mutant flies. n = 100 for each genotypes. Median survival for control male = 40days, *boss* mutant males = 13.5 days, control female = 33days, *boss* mutant females = 9days (Log-rank test, p<0.0001). (B) Analysis of vertical distance climbed in the rapid iterative negative geotaxis assay of *boss* mutant and control (w) male flies (n = 3 groups; each group contains 10 flies per genotype per time point). Data are means ± SEM, and differences are significant by t-test (*P<0.05).

To determine the role of BOSS in aging, we first examined the lifespan of *boss* mutant flies (Fig 1A). Both female and male *boss* mutant flies had shorter lifespans compared to control flies, indicating that aging in *boss* mutants might be accelerated.

To test the accelerated aging hypothesis, we used a behavioral task sensitive to age. Negative geotaxis is a natural escape response of flies to climb vertically, and it is an ability that declines with age [13, 14]. We compared age-related locomotor performance of mutant and control flies using the rapid iterative negative geotaxis assay [15], which objectively measures climbing performance by measuring negative geotaxis. We assessed performance of *boss* mutant and control flies of different ages: 1, 7, 14, 21, 28, and 35 days old. Young 1-day-old *boss* mutant flies displayed the same climbing performance as control flies, confirming normal locomotor development. However, performance declined more rapidly in *boss* mutant flies with age compared to control flies, with differences evident as young as 7 days old (Fig 1B). Additionally, the performance decline in control flies paralleled the survival rate, while in *boss* mutant flies the initial decline significantly exceeded the observed decline in survival rate. This hyperbolic decline characteristic in *boss* mutant flies can be interpreted as an increased population sickness already at a young age. Together, these results suggest that BOSS affects the lifespan of flies.

### Young *boss* mutant flies exhibit age-related decline of gut function

**Fig 2:**
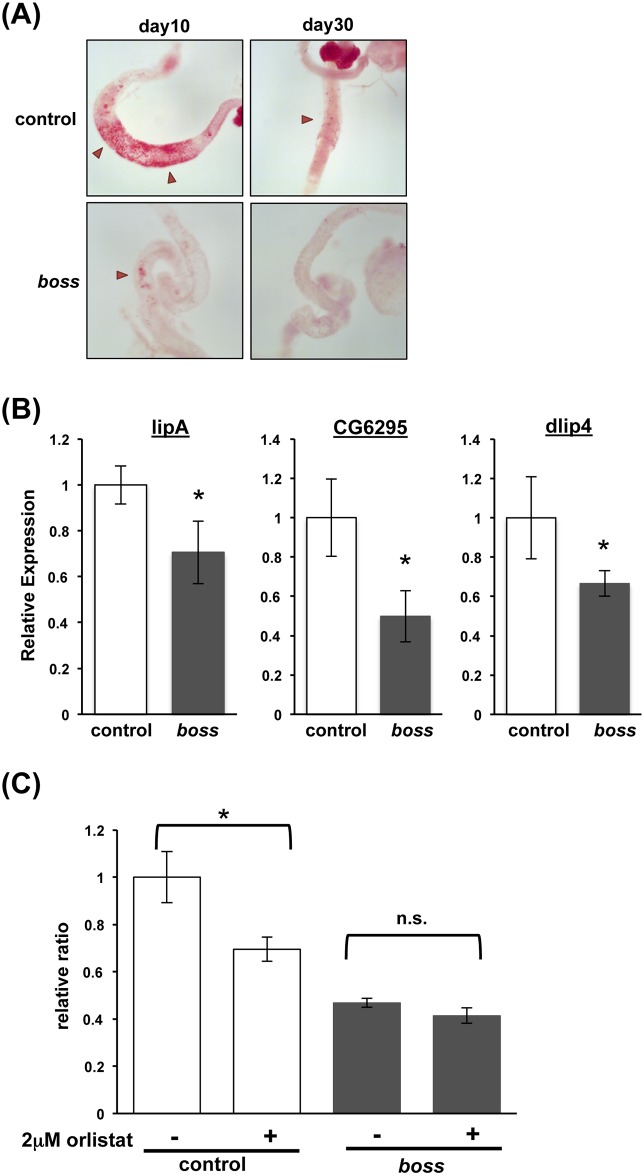
Gut lipase activity declines with aging in *boss* mutant flies. (A) Oil-Red-O staining of neutral lipids in the guts of young (10-days) and old (30-days) control and *boss* mutant female flies. Anterior midgut region stores lipid (arrowhead). (B) Expression of gut lipases (lipA/margo, CG6295, and dlip4) in the intestines of young (day 10) flies, as assessed by qRT-PCR (n = 3, 5 guts per replicate). (C) *boss* mutant flies are resistant to orlistat treatment. Young adult male *boss* mutant flies and control flies were fed a diet with or without orlistat (2.0 μM) for 5 days. Afterward TAG levels were determined (n = 5, 10 flies per replicate) and normalized for total protein. Levels are presented as normalized to levels for wild-type flies. Data are mean relative ratios ± SEM, and differences are significant by t-test (*P<0.05).

Next, we assessed gut lipase activity in *boss* mutant flies. Recently, it was reported that gut lipase activity decreases during aging [16, 17]. Gastric lipases digest lipids in food, which are then absorbed in the gut. Therefore, a reduction in gastric lipase activity decreases gut lipid storage. We found that gut lipid storage is reduced in young (10-day-old) *boss* mutant flies, similar to that in aged control flies (Fig 2A).

The expression of gastric lipases was also decreased in *boss* mutant flies. This is consistent with the decreased Oil-red-O staining in mutant flies (Fig 2A). Transcription of gastric lipases (lipA/margo, CG6295, and dlip4) was also significantly downregulated in *boss* mutant flies, as confirmed by qRT-PCR (Fig 2B).

To confirm and extend these results, we inhibited gastric lipases with orlistat (tetrahydrolipstatin). Orlistat is widely used as an over-the-counter weight-loss drug [18, 19]. It acts inside the intestine as a competitive inhibitor of pancreatic and gastric lipases, preventing their interaction with dietary triacylglycerides (TAG) and thus blocking fatty acid release and dietary lipid uptake. In control flies exposed to orlistat, TAG levels were significantly reduced (Fig 2C). Orlistat, however, failed to affect TAG levels in *boss* mutant flies, indicating that endogenous gastric lipase activity is very low. These data show that gastric lipase activity is decreased in *boss* mutant flies due to downregulation of gastric lipase expression.

Age-related chronic FOXO activation in enterocytes leads to sustained repression of gut lipase expression and disruption of lipid homeostasis [17]. We measured the expression of FOXO target genes (*Drosophila* insulin receptor [dInR] and Thor/4E-BP) [20], and found that dInR mRNA expression was significantly increased while Thor mRNA expression exhibited a slight, but statistically non-significant increase. This suggests that FOXO activation in the gut of *boss* mutant flies is enhanced (S1 Fig). Taken together, these data indicate that tissue homeostasis is disrupted in the gut of *boss* mutant flies similar to that in aged control flies.

### Oxidative stress is enhanced in *boss* mutant flies

Oxidative damage is a major risk factor for age-related diseases and aging. ROS, such as hydrogen peroxide (H_2_O_2_) and superoxide anions (O_2_^-^), are highly reactive molecules produced by incomplete reduction of oxygen, and their production causes oxidative damage [21]. Oxidative damage contributes to the onset of aging and to age-related diseases such as diabetes, obesity, and neurodegenerative disorders [21]. This prompted us to ask the question: Does oxidative damage accumulate more in *boss* mutant flies?

**Fig 3:**
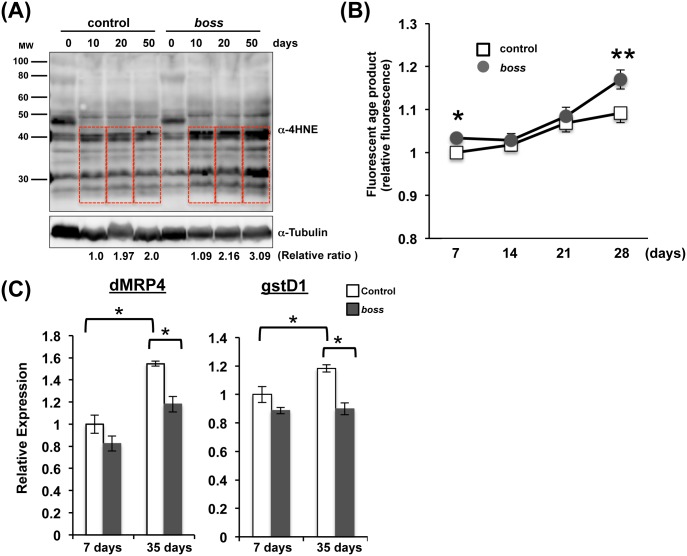
Oxidative stress is enhanced in *boss* mutant flies. (A) ALEs were detected by Western blot and antibodies against 4-hydroxy-2-nonenal (4HNE). Ages (0~50-days old) are shown at the top of the blot. (B) Accumulation of fluorescent AGEs was measured in control and *boss* mutant flies (n = 3, 20 flies per replicate). Data are mean relative fluorescence ± SEM, and differences are significant by t-test (*P<0.05, **P<0.01). (C) Expression of ROS-inducible genes (dMRP4 and gstD1) in young (7-days) and old (35- days) flies, assessed by qRT-PCR (n = 3, 10 flies per replicate). Data are mean relative expression ± SEM, and differences are significant by t-test (*P<0.05).

Since elevated ROS levels promote the oxidation of lipids and glucose, resulting in the accelerated formation of ALEs and AGEs [22, 23], we examined ALEs and AGEs levels to know oxidative levels in control and *boss* mutant flies. First, we measured the level of 4-hydroxy-2-nonenal (4-HNE), a major type of ALE. Under oxidative-stress conditions, lipid peroxidation of ω6-polyunsaturated fatty acids leads to the production of 4-HNE [24]. We found that 4-HNE levels were higher in *boss* mutant flies than in control flies at late stage (Fig 3A).

Next, we examined AGE levels during aging. In *Drosophila*, AGEs are also used as biomarkers of age-related damage [25]. AGEs fluoresce in a nonenzymatic reaction between reducing sugars and amine residues on proteins, lipoproteins, and nucleic acids. AGEs also accumulate during aging and correlate strongly with mortality rate in flies. We quantified fluorescent AGE levels in sets of young and old male flies during aging (7- to 28-days old). Fluorescent AGE levels were higher in *boss* mutant flies than in control flies at late stage (Fig 3B), indicating that oxidative stress was enhanced in *boss* mutant flies.

The elevated fluorescent AGE levels in *boss* mutant flies prompted us to examine the expression of marker genes (dMRP4 and gstD1) that mediate oxidative stress resistance (Fig 3C) [26, 27]. These genes are induced in adult flies during normal aging and when flies are subjected to oxidative stress, for example, by exposure to hyperoxic conditions or to paraquat. Both dMRP4 and gstD1 mRNAs were elevated in old control flies (35-days old) compared to young control flies (7-days old). However, dMRP4 and gstD1 mRNA expression was not elevated in old *boss* mutant flies, indicating that *boss* mutant flies are more sensitive to oxidative stress.

### *boss* mutant flies are sensitive to oxidative stress

Since oxidative stress is enhanced in *boss* mutant flies and expression of genes involved in resistance to it is reduced, we speculated that *boss* mutant flies might be more susceptible to conditions that increase oxidative stress. If this were the case, oxidative damage would accumulate faster as these flies age. To examine this hypothesis, we first measured *boss* mutant flies’ survival curve when subjected to oxidative stress.

**Fig 4:**
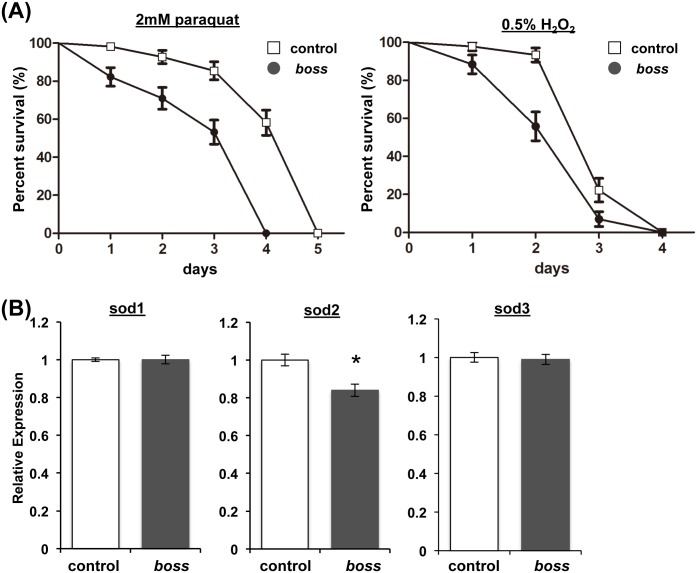
*boss* mutant flies are sensitive to oxidative stress. (A) Young adult male *boss* mutant and control flies were fed a diet containing either 2.0 mM paraquat or 0.5% H_2_O_2_, and survival rate of the groups was measured (n = 30 flies for each genotypes). Log-rank test, p<0.0001. Error bars represent S.E. (B) Expression of superoxide dismutases (SOD1-3) in young (10-days) flies as measured by qRT-PCR. Data are means ± SEM (t-test, *P<0.05).

*boss* mutant and control flies were given food containing either paraquat (2 mM) or H_2_O_2_ (0.5%). Under these conditions, *boss* mutant flies died more quickly than control flies (Fig 4A), providing more evidence that *boss* mutant flies are more sensitive to oxidative stress.

One reason to explain this increased susceptibility to oxidative stress is that levels of ROS in the mutant flies may be dysregulated. Cellular levels of ROS are controlled by antioxidant enzymes. Superoxide dismutase (SOD) is one of several major enzymes that regulate the removal of ROS. Similar to other metazoans, *Drosophila* has three SOD isoforms (SOD1-3) [28, 29]. SOD1 localizes to the cytoplasm, SOD2 to the in the mitochondrial matrix, and SOD3 to the extracellular space. Loss of either SOD1 or SOD2 causes early lethality in flies and mice [30, 31], whereas ubiquitous overexpression of either SOD1 or SOD2 extends lifespan [32–36]. This is in line with our findings that *boss* mutant flies have increased oxidative stress levels and increased sensitivity to oxidative stress.

We next measured the expression levels of SODs in *boss* mutant flies to test this idea. Quantitative RT-PCR revealed that expression of sod2 mRNA was decreased in *boss* mutant flies, while expression of sod1 and sod3 mRNA was comparable to that in control flies (Fig 4B).

The mitochondrion is the major organelle that produces ROS, and SOD2 is required to prevent increased ROS levels. Consistent with our findings and hypothesis is that *sod2* mutant flies have a shortened lifespan [37]. We speculated that decreased SOD2 level in *boss* mutant flies is one cause of their accelerated aging.

### SOD2 expression rescues sensitivity to oxidative stress of *boss* mutant flies

**Fig 5:**
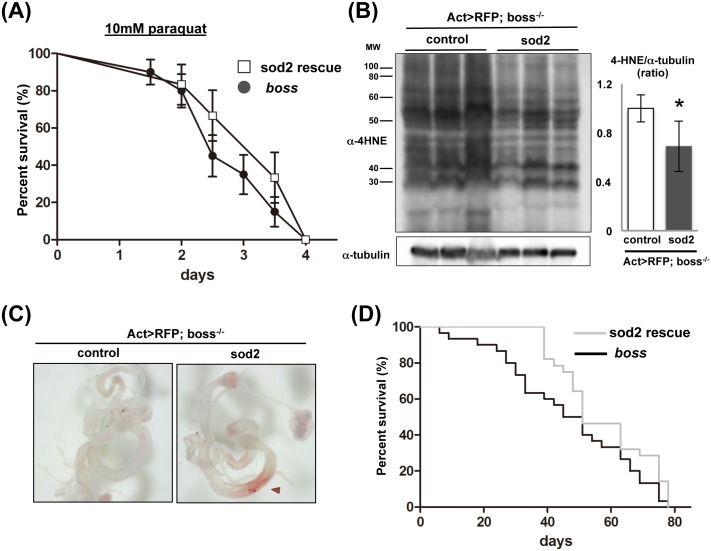
SOD2 overexpression rescues aging phenotype of *boss* mutant flies. (A) Survival curves of SOD2-overexpressing *boss* mutant flies (Act-Gal4, UAS-RFP/ UAS-SOD2; boss^-/-^) and *boss* mutant flies (Act-Gal4, UAS-RFP/ +; boss^-/-^) treated with 10 mM paraquat. SOD2 overexpression prolonged the survival of *boss* mutant flies, shifting the survival curve to the right of the population of SOD2-overexpressing *boss* mutant flies (n = 30 for each genotypes). Error bars represent S.E. (B) ALEs were detected by Western blot and anti-4HNE antibody. SOD2 overexpression reduced the staining intensity for ALEs (n = 3, 10 flies per replicate). (C) Oil-Red-O staining for neutral lipids in the guts of young (10-days) SOD2-overexpressing control and *boss* mutant female flies. Anterior midgut region stores lipids (arrowhead). (D) Lifespan for male SOD2-overexpressing *boss* mutant and *boss* mutant flies. SOD2 overexpression prolonged the survival of *boss* mutant flies. Survival was presented by Kaplan Meier curves of SOD2-overexpressing *boss* mutant and *boss* mutant flies. n = 50 for each genotypes. Median survival for SOD2-overexpressing *boss* mutant = 51 days, *boss* mutant males = 48 days (Log-rank test, p = 0.044).

To assess the role of SOD2 in *boss* mutant flies, we generated SOD2-overexpressing *boss* mutant flies by crossing Act-Gal4 flies with UAS SOD2 flies. This resulted in Act-GAL4/UAS-SOD2 flies with a boss mutant background (S2 Fig). First, we examined whether these flies are sensitive to oxidative stress by exposing them to paraquat (10 mM) in their feed, as before (Fig 5A). Under this condition, SOD2-overexpressing *boss* mutant flies lived longer than *boss* mutant flies, supporting our hypothesis that decreased SOD2 levels in *boss* mutant flies is one cause of their accelerated aging under oxidative stress-producing conditions.

Next, we assessed oxidative stress levels in SOD2-overexpressing *boss* mutant flies by measuring 4-HNE levels (Fig 5B). SOD2 overexpression reduced 4-HNE levels. These results demonstrate that sensitivity to oxidative stress was rescued by SOD2 overexpression in *boss* mutant flies.

We also examined gut lipid storage in SOD2-overexpressing *boss* mutant flies (Fig 5C). The decreased Oil-red-O staining we observed earlier in young *boss* mutant flies (cf. Fig 2A) was recovered in SOD2-overexpressing *boss* mutant flies. This suggests that SOD2 expression can rescue gastric lipase activity of boss mutant flies. This is also in line with the observation that SOD2 overexpression increases the lifespan of *boss* mutant flies under normal conditions (Fig 5D). From these SOD2 rescue experiments, we conclude that decreased SOD2 expression in *boss* mutant flies contributes to their shortened lifespan.

### Regulation of mitochondrial activity is disrupted in *boss* mutant flies

We previously reported that energy state is reduced in *boss* mutant flies [11, 12]. Reducing cellular energy levels by starvation or exercise increases mitochondrial activity, and hence production of the energy source, ATP[9,38]. This led us to speculate that mitochondrial mass increases in *boss* mutant flies in order to meet energy demand.

**Fig 6:**
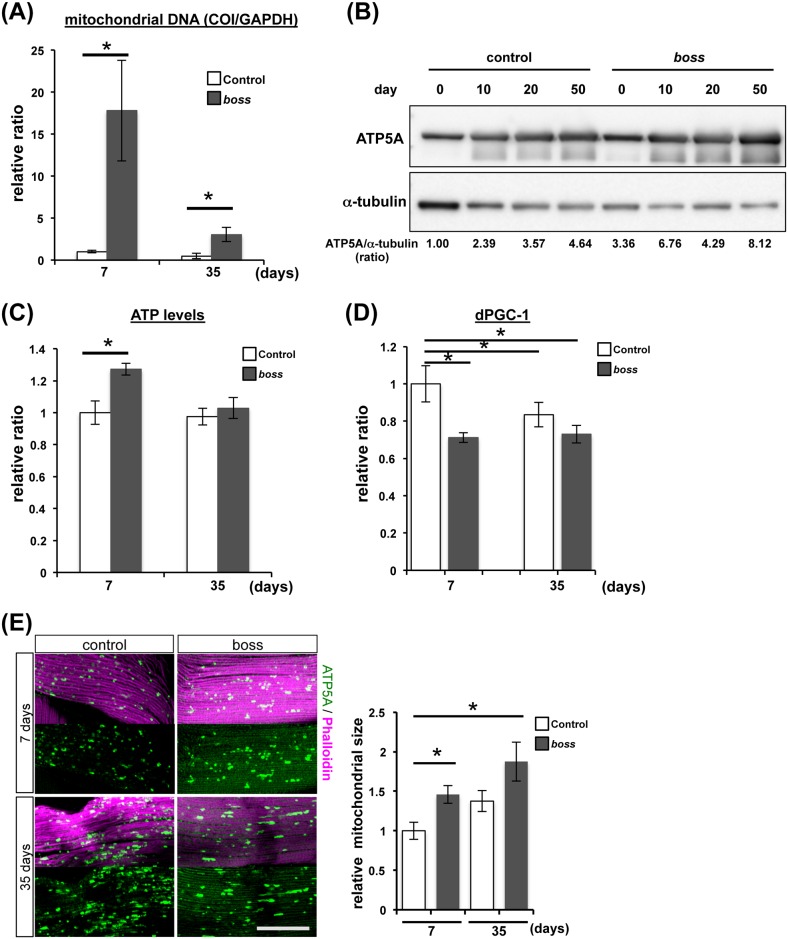
Mitochondrial mass is increased in *boss* mutant flies. (A) Quantification of mitochondrial DNA (mtDNA) in control and *boss* mutant flies as determined by qPCR (n = 3, 5 flies per replicate). mtDNA of both young (7-days) and old (35-days) *boss* mutant flies was greater than in control flies. Data are means ± SEM (t-test, *P<0.05). (B) The amount of mitochondria was determined using Western blotting and anti-ATP5A antibody. ATP5 levels were increased in *boss* mutant flies, suggesting that the total mass of mitochondria increased. Values at bottom of columns are quantitation of ATP5A densitometry analysis (ATP5A/tubulin ratio). (C) ATP production levels were measured in young (7-days) and old (35-days) flies (n = 3, 5 flies per replicate). Data are means ± SEM (t-test, *P<0.05). (D) Expression of *pgc1* mRNA was measured in young (7-days) and old (35 days) flies by qRT-PCR (n = 3, 10 flies per replicate). *pgc-1* mRNA levels were significantly decreased in young *boss* mutant flies, indicating indicates mitochondrial biogenesis is reduced. Data are means ± SEM (t-test, *P<0.05). (E) Representative confocal images showing indirect flight muscles stained with phalloidin (magenta) and anti-ATP5 antibody (green). One representative optical section is shown from each phenotype. Scale bar, 50μm. Comparison of the mitochondrial size of both young (7-days) and old (35-days) of control and *boss* mutant flies are shown to the right. Data are means ± SEM (t-test, *P<0.05).

To determine mitochondrial mass as a proxy measure, we measured the amount of mitochondrial DNA (mtDNA) relative to the amount of nuclear DNA (nDNA) in young (7-days old) and old (35-days old) flies. The amount of mtDNA increased in *boss* mutant flies (Fig 6A). We also examined the expression of ATP5A, a mitochondrial marker, in order to monitor the amount of mitochondria in flies. ATP5A levels were higher in *boss* mutant flies compared to control flies (Fig 6B), which is consistent with our results on mtDNA quantification (c.f. Fig 6A).

Next, we measured ATP levels in control and *boss* mutant flies, since mitochondria is at the core of cellular energy metabolism, being the site of most ATP generation but also responsible for generation of substantial amounts of superoxide caused by electron leakage from the oxidative phosphorylation pathway [7, 38]. ATP concentrations were increased in young *boss* mutant flies, indicating that mitochondrial activity is upregulated in *boss* mutant flies; ATP concentrations were the same in old control and *boss* mutant flies (Fig 6C). The increased mitochondrial mass without increased ATP production indicated that unfunctional mitochondria are accumulated in old boss mutant flies.

Mitochondria are energy-converting organelles that produce most of the ATP required for cellular functions and integrity, and they produce ROS as byproducts of electron transport during the generation of ATP. ROS cause mtDNA mutations to accumulate, which may be responsible for a decline in mitochondrial energy production. Thus, cells have developed mechanisms—or mitochondria “quality control system”—to eliminate damaged mitochondria [21].

The increased mitochondrial mass in *boss* mutant flies may be due to dysregulation of this mitochondrial quality control system. To test this possibility, we examined the expression of a key molecule in this system called peroxisome proliferator-activated receptor gamma co-activator 1 (PGC-1), a transcription coactivator. PGC-1 regulates gene expression for mitochondrial biogenesis and maintains the structural integrity of mitochondria [39, 40]. *PGC-1* mRNA levels in control and *boss* mutant flies at young and old time points were compared (Fig 6D). We compared PGC-1 mRNA levels in young and old control and *boss* mutant flies, and found that PGC-1 mRNA levels were significantly decreased in young *boss* mutant flies. This indicates that mitochondrial biogenesis is reduced (Fig 6D). Mitochondrial mass was increased throughout the lifespan of *boss* mutant flies. However, increased ATP levels were present only in young flies, strongly indicating that dysfunctional mitochondria accumulate as *boss* mutant flies age. In *Drosophila*, size of mitochondria is increased during aging[41]. This is consistent with our finding that mitochondria in the flight muscles of young (7-days old) and old (35-days old) *boss* mutant flies were much larger than those in comparably aged control flies (Fig 6E). Taken together, the age-related increase in mtDNA and mitochondrial size with a concomitant decrease in ATP levels might point towards reduced mtDNA quality in aging *boss* mutants.

### Hsp22 expression is not increased in *boss* mutant flies during aging and oxidative stress stimulation

**Fig 7:**
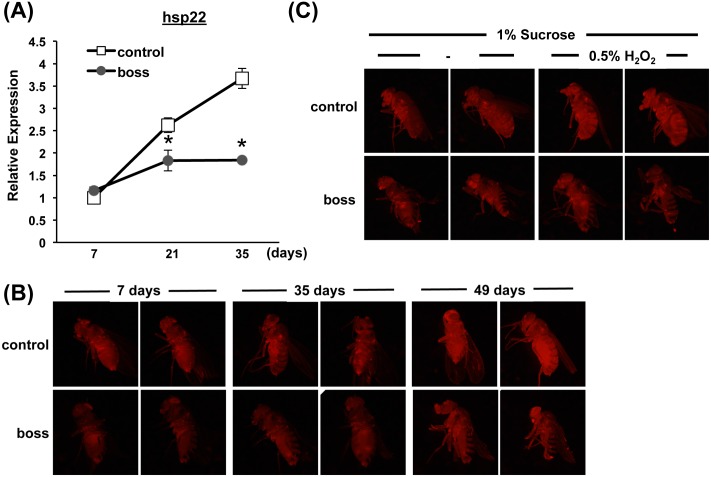
hsp22 expression is not induced in *boss* mutant flies. (A) Expression of *hsp22* mRNA was measured in 7-, 21-and 35-days-old control and *boss* mutant flies by using qRT-PCR (n = 3, 10 flies per replicate). Data are means ± SEM (t-test, *P<0.05). (B) DsRed fluorescence was observed in control and *boss* mutant flies bearing the Hsp22-DsRed reporter. DsRed fluorescence progressively increased during aging in control but not in *boss* mutant flies. (C) Flies bearing the Hsp22-DsRed reporter were transferred to a diet containing 0.5% H_2_O_2_ for 24 h, and then DsRed fluorescence was observed. Under this oxidative stressful condition, *boss* mutant flies still failed to exhibit increased *hsp22* expression, suggesting dysfunctional mitochondria and oxidative stress dysregulation.

Finally, we examined *hsp22* mRNA expression levels. Hsp22 belongs to the sHSP family, localizes in the mitochondrial matrix, and is involved in aging processes, and imparts resistance to oxidative stress [42, 43]. HSP22 expression is upregulated during aging [44], in a long-lived fly line [45], and in response to oxidative stress [27]. Corroborating a previous report [44], *hsp22* expression progressively increased with aging in control flies (Fig 7A). However, while *hsp22* expression was indistinguishable in young (7-days old) control and *boss* mutant flies, expression diverged with aging between the two groups. In *boss* mutant flies aged 21and 35 days, *hsp22* expression was significantly lower in the older flies.

We also examined hsp22 levels a second way, using *hsp22* reporter (hsp22-dsRED) [44]. The promoter sequence of *hsp22* gene is fused to DsRED; thus, DsRED expression level reflects *hsp22* expression level. hsp22-DsRED expression increased with age in wild-type flies but not in *boss* mutant flies (Fig 7B). Oxidative stress stimulation with H_2_O_2_ also clearly increased Hsp22-dsRED expression in wild-type flies but slightly in *boss* mutant flies (Fig 7C), supporting the notion that *boss* mutant flies apparently cannot respond adequately to oxidative stress, and thus fail to maintain mitochondrial integrity.

## Discussion

Aging is associated with a decline of function at the organismal level. It begins with cellular deterioration, which ultimately leads to the disruption of tissue homeostasis. The present study indicates that BOSS influences on aging. We showed that *boss* mutant flies have a shortened lifespan (Fig 1), which is caused by deterioration of gut homeostasis and by elevation of oxidative stress. Even in young *boss* mutant flies, gastric lipase expression and activity are already reduced (Fig 2). Enhanced oxidative stress was also observed in *boss* mutant flies (Fig 3), and this was caused by an impaired ROS defense system (Figs 4–6). Our genetic analysis of SOD2 expression rescue showed that reducing oxidative stress could rescue all aged phenotypes of the *boss* mutant (Fig 5).

We previously reported that energy metabolism is disrupted in *boss* mutant flies, causing lean phenotype [11, 12]. The gut is one of the key tissues for aging regulation [17]. Hyper-proliferation of intestinal stem cells (ISCs) occurs in the aged gut, resulting in loss of tissue homeostasis, elevated oxidative stress, and chronic JNK activation [16, 46, 47]. In *boss* mutant flies, we found that oxidative stress is increased (Fig 3) and gut homeostasis is impaired (Fig 2). Moreover, the expression and activity of gastric lipases were decreased in *boss* mutant flies, producing a similar phenotype to that observed in aged control flies. Previously, we demonstrated that BOSS is expressed in enteroendocrine cells, and knockdown of *boss* in these cells lead to the lean phenotype [12]. Thus, it would be interesting to know whether BOSS expressed in enteroendocrine cells and/or in other tissues (such as neurons and the fat body) affect gut homeostasis.

A number of recent studies indicate that regulation of ROS levels seems to be very important for aging. ROS is concomitantly generated during ATP production and causes oxidative damage and cellular/tissue dysfunction if it accumulates [4]. Excessive ROS levels have a harmful effect on cellular physiology [31, 37, 48], whereas physiological levels of ROS are likely essential for the maintenance of cellular homeostasis [49]. This is called mitohormesis or adaptive cytoprotective responses to low levels of oxidative stress in the mitochondria [50, 51]. Thus, disrupted mitohormesis affects disease onset, progression, and aging.

Mitochondria are energy-converting organelles that produce most of the ATP required for cellular functions and integrity, and they produce ROS as byproducts of electron transport during the generation of ATP. ROS cause mtDNA mutations to accumulate, which may be responsible for a decline in mitochondrial energy production. Thus, cells have developed mechanisms—or mitochondria “quality control system”—to eliminate damaged mitochondria [21]. In the present study, ATP production was increased in young *boss* mutant flies, and mitochondrial mass was increased throughout their life (Fig 6). This indicates that dysregulation of mitochondrial homeostasis lead to loss of tissue homeostasis. Indeed, we observed that the expression of *hsp22*—which localizes in mitochondria and activates the ROS defense system (sod2, dMRP4, and gstD1)—was decreased in *boss* mutants (Fig 7). Aged phenotypes were also rescued by *sod2* overexpression (Fig 5). Finally, we found that PGC-1 expression was decreased in *boss* mutant flies (Fig 6D).

Since PGC-1 and Hsp22 increase mitochondrial biogenesis and extend lifespan in *Drosophila* [43, 47], we speculate that the acceleration of aging seen in the *boss* mutant flies is likely caused by disruption of mitochondrial homeostasis. We previously found that *boss* mutants are hyperphagic but paradoxically are lean, indicating that nutrient absorption might be disrupted [12]. If this were the case, more mitochondria would be required to generate more ATP. Thus, we speculate that the number of mitochondria and their activity might be increased in *boss* mutant flies. However, concomitant generation of ROS might lead to cellular damage and loss of metabolic homeostasis, ultimately leading to accelerated aging in *boss* mutant flies.

Our data strongly indicate that increased ROS levels influence the aging process, and that normal mitochondrial function and structure are also damaged in the *boss* mutant. Very recently, it was proposed that mitochondria affect the aging process not only by producing ROS but also via other mitochondrial signaling pathways. Thus, changes in mitochondrial dynamics and the electron transport chain could have an impact on aging [7, 52]. It would be interesting and important, then, in the future to examine the molecular mechanism of how BOSS localized in cell membranes regulates mitochondrial function and structure.

BOSS is an evolutionarily conserved protein [53]. The expression of GPRC5B, a mammalian BOSS homologue, is similar to that of BOSS [54–56]. GPRC5B is broadly expressed in brain, gut, and adipose tissues. Recently, aberrant expression of GPRC5B was identified to be an obesity risk factor [57]. Considering that the regulatory mechanisms of energy metabolism and mitochondrial quality control are highly conserved between *Drosophila* and mammals [58, 59], specific analysis of *Drosophila* BOSS will open the door for understanding the broader physiological functions of the highly conserved membrane protein BOSS/GPRC5B.

## Materials and Methods

### Fly stocks and genetics

The following fly strains were used: w (WT, control); *boss*^1^ (null mutant, homozygous viable), Act-Gal4>UAS-RFP (Kyoto Stock Center, DGGR#4414), UAS-Sod2 (Bloomington Drosophila Stock Center, #24494); hsp22-RED (a kind gift from Dr. John Tower).

### Husbandry

Flies were grown on standard food (10% glucose/4% yeast/4% corn meal/1% agar) at a temperature of 25°C under a 12h-12h light-dark regime.

### Lifespan analysis

Female or male flies were collected at day 0 and maintained at a density of 10 or 20 flies per vial. Flies were transferred to new vials every 3 days, and dead ones were noted in order to calculate survival curves.

### Rapid iterative negative geotaxis assay

Fly climbing speed was assessed using the RING assay, as described previously [15]. 10 flies (n = 3 groups) were transfer into each vial without anesthetizing. The vials were tapped and all flies were knocked down to the bottom of each vial. After 3 seconds a picture was taken to record the climb height. The procedure was repeated 5 times at 1min intervals and the average height climbed of each vial was scored.

### Quantitative real-time PCR (qRT-PCR)

Guts of female flies, aged 5 days, were used for RNA samples to measure gastric lipase expression (Fig 2B). To measure SOD1~3 expression (Fig 4B and S2 Fig) were collected from whole flies. Samples were immediately frozen in liquid nitrogen for later analysis. Total RNA was extracted using Trizol Reagent (Invitrogen). Total RNA samples (1 μg per reaction) were reverse transcribed using oligo-dT and random primers and Superscript RT-III (Invitrogen). The generated cDNA was used for real-time RT-PCR (qPCR Mastermix Plus for SYBERGreen; Applied Biosystems). Rp49 gene was used as reference gene. Two-step RT-PCR was performed with the following condition: denature temperature was 95°C and anneal and extend temperature was 60°C. Three separate samples were collected from each condition, and triplicate measurements were made. Primers (S1 Table) were designed using the Universal Probe Library Assay Design Center (Roche Applied Science).

### Western blot analysis

Ten male flies were used for each condition. The antibodies used in Western blot analysis were as follows: anti-4HNE (1:1000, JalCA); anti-ATP5A (1:100,000, Abcam); and anti-tubulin (1:10,000, Sigma). HRP-conjugated anti-rabbit or anti-mouse IgG antibodies (Cell Signaling Technology) were used as secondary antibodies. Blots were visualized using Chemi-Lumi One Super reagent (Nacali Tesque Inc.). To quantify 4-HNE and ATP5A levels, staining intensity of each lane was measured using ImageJ (http://imagej.nih.gov/ij/). Measured area is indicated in the box.

### Oil-Red-O staining

Guts from female adult flies, aged 10 and 30 days, were fixed in 4% paraformaldehyde for 25 min at room temperature, washed with distilled water, and incubated in 100% propylene glycol for 5 min. Specimens were then incubated at 60°C in Oil-Red-O stain (0.5% Oil Red O in propylene glycol), washed twice with propylene glycol, washed three times with PBS, and mounted onto glass slides in 20% glycerol/PBS for imaging.

### TAG assay

Orlistat treatment procedures involved growing flies on standard food with or without 2 μM orlistat for 5 days. TAG levels were then assayed. For the TAG assay, male flies aged 3–5 days were homogenized in PBS. Supernatant was collected after heat inactivation at 70°C for 5 min, and centrifugation at 13000 rpm for 5 min. TAG was measured using a triglyceride assay kit (Sigma). Data were normalized to total protein.

### Oxidative stress assay

To induce oxidative stress, we fed male flies aged 5 days with standard food with 0.5% H_2_O_2_ or 2 mM paraquat (Fig 4A) or 10mM paraquat (Fig 5A). The number of living flies were noted every day and the survival rate was calculated.

### Fluorescent advanced glycation end products (AGEs)

Fluorescent AGEs were measured as previously described [22]. Twenty male flies were homogenized in 900 μl of 10 mM EDTA-Na_2_.H_2_O in PBS. Homogenate was transferred to a microcentrifuge tube containing 100 μl of a solution containing 10 mg trypsin/10 mM EDTA-Na_2_.H_2_O in PBS. Following incubation for 24 h at 37°C, the digested homogenate was centrifuged at 11,000g for 5 min. The supernatant was spin-filtered through a 0.22 μm cellulose membrane (Millipore). Aliquots of the filtrate (200 μl) were transferred to 96-well plates, and fluorescence was measured at excitation and emission wavelengths of 365 nm and 440 nm, respectively. Fluorescence values for analysis were taken as the mean fluorescence from triplicate wells.

### Mitochondrial DNA measurement

Mitochondrial DNA content was determined by the ratio of the gene for cytochrome oxidase subunit I [57] to a nuclear gene GAPDH (S1 Table). Total DNA from the whole flies (n = 3, 20 flies per replicate) were prepared by homogenization in 10 mM Tris-HCl, pH 8.0, 1 mM EDTA, 0.1% Triton X-100 and 10μg/ml protease K. Following 60 min incubation at 37°C, Protease K was heat inactivated at 95°C for 5 min. Mitochondrial DNA was quantified relative to nuclear DNA by the ratio of cytochrome oxidase subunit I [57] to glyceraldehyde 3-phosphate dehydrogenase (GAPDH) in quantitative real-time PCRs. DNA copy numbers were measured by using real-time PCR as described above.

### ATP quantification

ATP levels were detected according to the procedures detailed in Tennessen et al. [60]. Five 10-day-old adult flies were used for this assay in the present experiment. Cellular ATP content was quantified with a luciferin/luciferase-based assay using an ATP Determination Kit (Sigma-Aldrich), and the data were normalized to the protein content.

### Immunohistochemistry

7-day-old and 35-day old adult flies were used for the flight muscles staining with anti-ATP5A (1:10,000, Abcam). To measure mitochondrial dimensions, the particle analysis function of ImageJ was used.

## Supporting Information

S1 Fig Transcription of Foxo target genes *thor* and *dInR* in the guts of wild-type and *boss* mutant flies. Expression of thor and dInR mRNAs was measured in young (7-days old) flies by qRT-PCR (n = 3, 10 flies per replicate).
(TIF)

S2 Fig Quantification of sod2 mRNA expression. Expression of sod2 mRNA was measured in young (7-days old) flies by qRT-PCR (n = 3, 10 flies per replicate). Data are means ± SEM (*P<0.05).
(TIF)

S1 Table Primer sequences for qRT-PCR. (TIF)
