# Supplementary figures and images for: Loss of BOSS Causes Shortened Lifespan with Mitochondrial Dysfunction in Drosophila
Source: PLoS One. 2017 Jan 3;12(1):e0169073. doi: 10.1371/journal.pone.0169073 (PMC5207625; doi:10.1371/journal.pone.0169073)

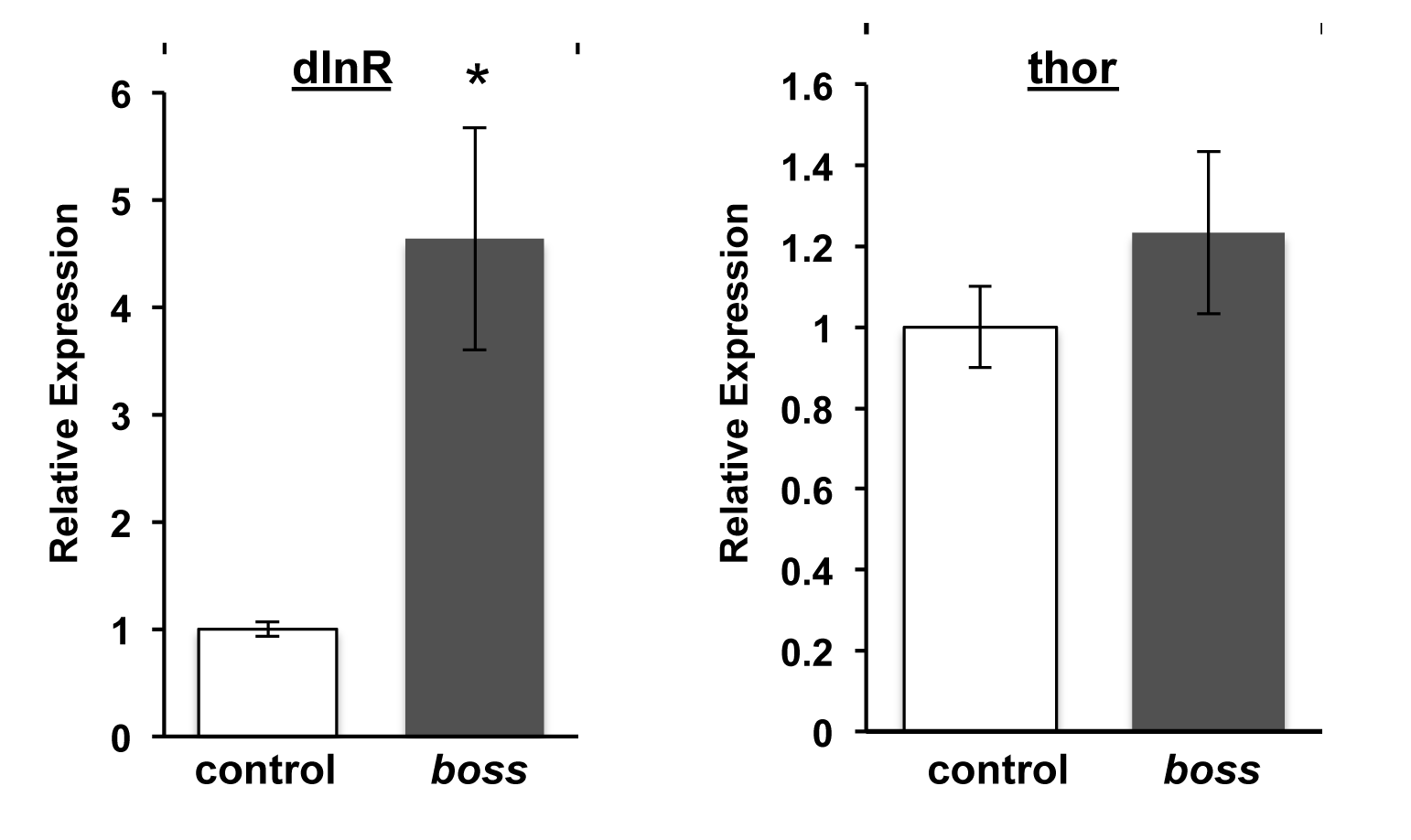

Supplement: S1 Fig — Expression of thor and dInR mRNAs was measured in young (7-days old) flies by qRT-PCR (n = 3, 10 flies per replicate). (TIF) [file pone.0169073.s001.tif]

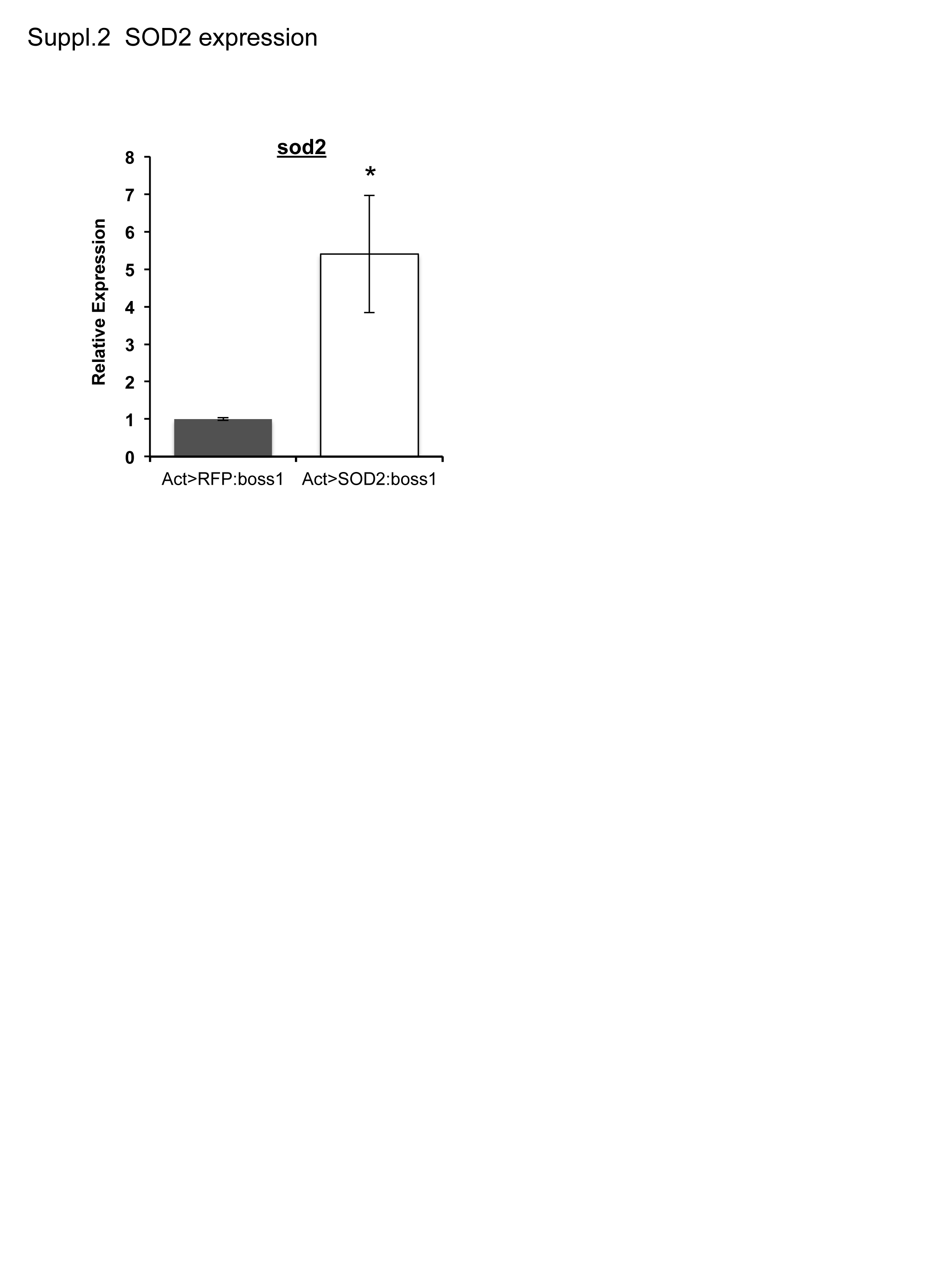

Supplement: S2 Fig — Expression of sod2 mRNA was measured in young (7-days old) flies by qRT-PCR (n = 3, 10 flies per replicate). Data are means ± SEM (*P<0.05). (TIF) [file pone.0169073.s002.tif]

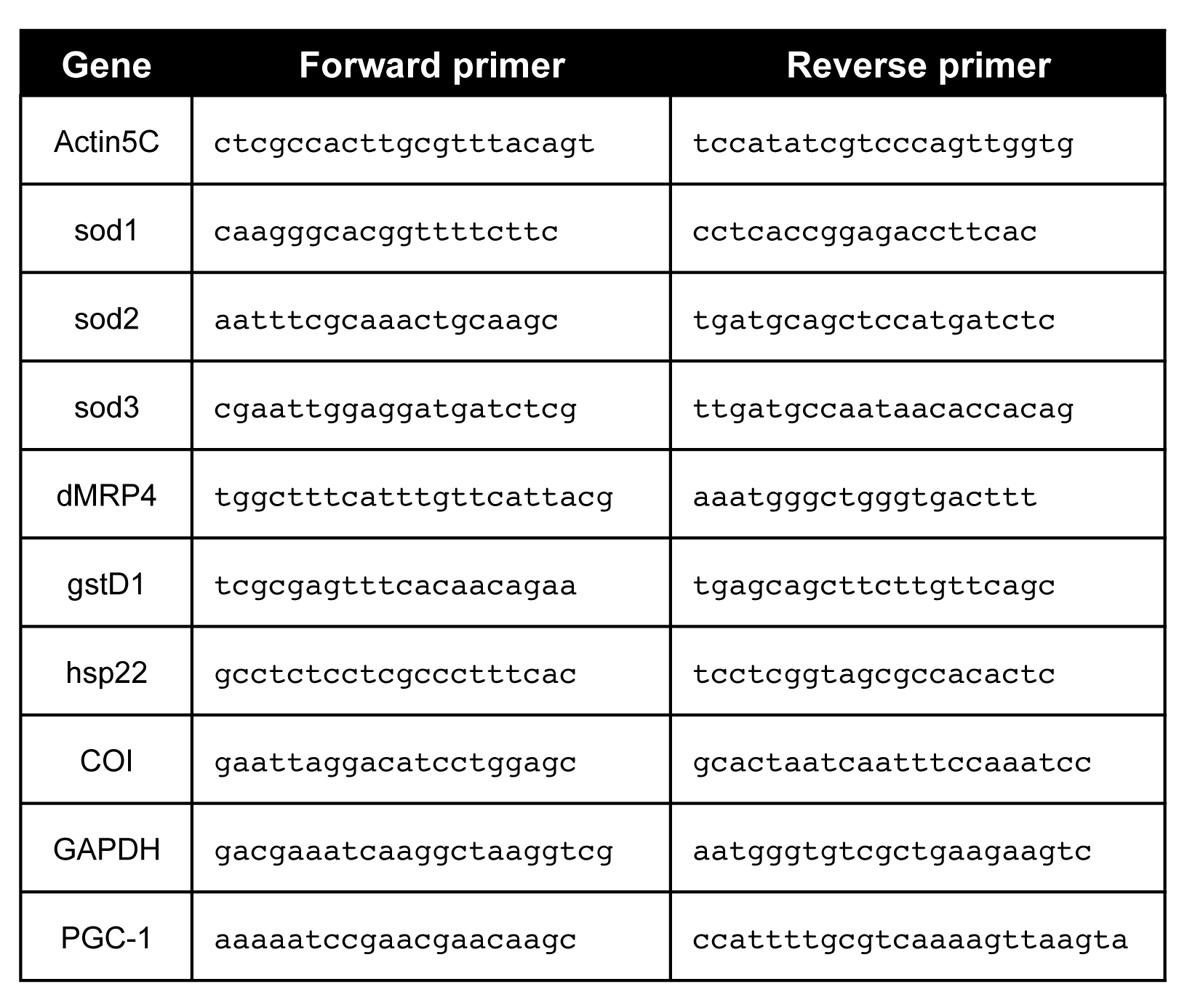

Supplement: S1 Table — (TIF) [file pone.0169073.s003.tif]
